# Food Seeking in a Risky Environment: A Method for Evaluating Risk and Reward Value in Food Seeking and Consumption in Mice

**DOI:** 10.3389/fnins.2017.00024

**Published:** 2017-01-30

**Authors:** Sarah H. Lockie, Clare V. McAuley, Sasha Rawlinson, Natalie Guiney, Zane B. Andrews

**Affiliations:** Monash Biomedicine Discovery Institute and Department of Physiology, Monash UniversityClayton, VIC, Australia

**Keywords:** behavior, food seeking, open field test, light dark box test, reward, risk

## Abstract

Most studies that measure food intake in mice do so in the home cage environment. This necessarily means that mice do not engage in food seeking before consumption, a behavior that is ubiquitous in free-living animals. We modified and validated several commonly used anxiety tests to include a palatable food reward within the anxiogenic zone. This allowed us to assess risk-taking behavior in food seeking in mice in response to different metabolic stimuli. We modified the open field test and the light/dark box by placing palatable peanut butter chips within a designated food zone inside the anxiogenic zone of each apparatus. We then assessed parameters of the interaction with the food reward. Fasted mice or mice treated with ghrelin showed increased consumption and increased time spent in the food zone immediately around the food reward compared to *ad libitum* fed mice or mice treated with saline. However, fasted mice treated with IP glucose before exposure to the behavioral arena showed reduced time in the food zone compared to fasted controls, indicating that acute metabolic signals can modify the assessment of safety in food seeking in a risky environment. The tests described in this study will be useful in assessing risk processing and incentive salience of food reward, which are intrinsic components of food acquisition outside of the laboratory environment, in a range of genetic and pharmacological models.

## Introduction

In a free-living situation, organisms always face a trade off in procurement of food. They must evaluate the risk of obtaining food in an unsafe environment, against the reward value of the food itself once obtained. This calculation must necessarily be dynamic and quickly recalculated when changes to either the environment or the nutritional needs of the organism occur. This flexible value attached to the food reward can be termed the “incentive salience” of the reward. The incentive salience (i.e., the importance) of the food is heavily influenced by physiological state (Lockie and Andrews, 2013). Studies of feeding behavior in laboratory animals, generally focused on mice, do not tend to take this balance into account, with most relying on a simple measurement of food consumed in the safe home cage environment. This largely removes the food-seeking behavioral component of food consumption, which is ubiquitous in animals outside of those kept as pets. Even humans in the current “obesigenic” environment must engage in food seeking behavior with some inherent risk, such as driving to the grocery store or restaurants. Studies that measure only food intake in a cage may miss deficits in the complete feeding behavioral spectrum, as they are not equipped to measure them. We have set out to address this issue by establishing and optimizing a set of simple and cost-effective behavioral tasks, using traditional behavioral equipment, designed to evaluate the incentive salience of a food reward in a risky environment.

Previous models of risk have approached this idea differently, using an operant conditioning paradigm and either a variable reward schedule or choice between a small, safe reward, and a large, “risky” reward—where “risky” is defined as an intermittent reward schedule for that lever (Leblond et al., 2011). This falls short of modeling “risk” as loss of a potential reward does not result in punishment or loss of personal resources. This task was redesigned to include a possible foot shock delivered along with the food reward (Simon and Setlow, 2012). These variations are informative regarding decision making in reward seeking, but fall short of modeling environmental risk *per se* as the animal can choose to avoid the risky lever entirely. Current mouse risk/reward paradigms do not incorporate the intrinsic risk associated with reward or food seeking in natural environments, such as injury and predation risk (Anselme, 2015). Others have previously used a modified light/dark box task with food included in the light zone (Teegarden and Bale, 2007; Cottone et al., 2012; Liu et al., 2016) as a measure of food seeking in a risky environment, and in this paper we seek to expand, validate and standardize this idea. Considering the light/dark box, like most common tests of unconditioned anxiety, relies upon the evolutionarily conserved fear of predation (Crawley, 2006), we supposed that this could be exploited to model ecologically relevant risk processing in food-seeking behavior in mice.

We have modified several standard behavioral tests of anxiety-like behavior to include a food reward element in the anxiogenic zone. We have experimentally validated these tests in a number of paradigms designed to alter the motivational state of the animal and/or the incentive salience of the reward. We fasted mice for 16 h or administered exogenous ghrelin, as both interventions are well-known to enhance food consumption, food seeking, and lever pressing in operant conditioning tasks (Tang-Christensen et al., 2004; Overduin et al., 2012; Skibicka et al., 2012). To examine the tests' sensitivity to acute metabolic state, we also included a group that had been fasted for 16 h and then administered IP glucose. Previous studies have shown that glucose can inhibit feeding in fasted mice, and that blood glucose levels can influence reported hunger levels in humans (Mayer, 1953; Bady et al., 2006), so we hypothesized that these mice should behave like fed mice.

## Methods

### Animals

C57black/6 male mice were obtained from Monash Animal Services at 8 weeks of age. For modified light/dark box and open field they were housed in pairs at 22°C in a 12:12 light/dark cycle. They were fed standard laboratory chow (Specialty Feeds, Glen Forrest, WA). Palatable food rewards were Reece's peanut butter chips (The Hersey Company, Hershey, PA, USA), referred to throughout as PB chips. All mice were exposed to the PB chips three times in the home cage before the test day to avoid neophobia and to familiarize mice with the taste and caloric content of the chips. All experiments were performed in the last 4 h of the light phase. For fasting experiments, food was removed at 1 h after the onset of the dark on the previous day and testing was conducted in the last 4 h of the light cycle on the test day. Food was returned immediately upon completion of the behavioral task.

### Drugs

Ghrelin (Rat, SC1356, lot number HF40076B, PolyPeptide group, Strasbourg, France) was administered at 0.3 mg/kg, in saline, and injected IP at 10 ml/kg. This dose was previously determined by dose response to be the lowest to reliably increase feeding in a 2 h window (Lockie et al., 2015). Ghrelin or saline vehicle were given 5 min before mice were placed in the apparatus. Glucose was given at 2.25 g/kg in 10 ml/kg of distilled water, not saline, to limit hypertonicity of the injection. Control mice for glucose injections received saline, not distilled water, avoid hypotonicity of the injection. Glucose was given 10 min before mice were placed in the apparatus.

### Behavioral tasks

#### Modified open field

The open field arena consisted of a circular area with a larger than normal diameter of 800 mm to increase the open field and enhance anxiety. PB chips were placed in an equilateral triangle within the center zone of the open field, with each PB chip being 280 mm from the external wall. PB chips of known weight were secured to the floor of the arena with Blu-Tack (Bostic). All other parameters were as per usual open field protocols, as follows. After injections, each mouse was placed in the same area of the perimeter of the open field and allowed to explore the space without interruption for 10 min. The trial was filmed for later analysis. After 10 min, the mouse was removed from the arena and placed back in the home cage with *ad libitum* access to food and water. The PB chips were removed and weighed and the apparatus cleaned with mild soap and water and allowed to dry.

#### Modified light/dark box

The light dark box apparatus consisted of a two-chambered box, with a large, white zone (480 × 300 mm) and a smaller black zone (150 × 300 mm). Both zones were open to the light at the top. A single, previously weighed PB chip was secured in the center of the center of light zone with Blu-Tack, 240 mm from the entry to the dark zone. Mice were injected, then placed in the dark side of the apparatus. They were allowed 10 min to explore the apparatus undisturbed, and trials were taped for later analysis.

#### Modified elevated plus maze

The elevated plus maze apparatus was a standard mouse plus maze with two open and two closed arms, with each arm being 50 × 300 mm, and a center zone 50 × 50 mm. Each open arm was baited with a PB chip 150 mm from the center zone, which was fixed to the arm with Blu-Tack. Mice were placed in the center zone facing the same open arm each time. They were allowed to explore the apparatus for 6 min and trials were filmed for later analysis.

#### Video analysis

All behavior tests were filmed and video analysis was done later using CleverSys TopScan software. Zoning for software analysis included a “food zone,” which was the area immediately surrounding the PB chip. For images of sample arenas, see Supplementary Figure 2. The food zone was always 78 mm in diameter, with the PB chip at the center of the zone. Mice were tracked from the center of the body and time in food zone was defined as center of the body within the 78 mm zone. Individual bouts were defined as the mouse entering and then leaving the zone, with the length of the bout being defined as the amount of time the mouse was continuously tracked within that zone. Latency was defined as time to approach one of the PB chips after introduction into the arena. For the reward-baited open field, approach of any one of the three pellets was considered the initial entry into the food zone.

#### Validation parameters and statistics

We were aiming to see data variation consistent with our results from standard versions of open field, elevated plus maze, and light/dark box, typically needing around 15/group to achieve an alpha of <0.05 for an effect size of ~0.8. We required the tests to be similarly sensitive to the non-modified open field, light dark box, and elevated plus maze. We intend to use these tests to screen genetically modified mice, and so we require a large to very large effect size to minimize animal numbers used in these tests, meaning values of Cohen's *d*-values of 0.8–1.2. Accepting an α of 0.05 and a β of 0.8, we need a sample size in the range of 10–21 mice/group, calculated using G^*^power (Faul et al., 2007). All statistics were performed using Graphpad prism software. Independent measures *t*-tests were used to compare results, with an alpha level of <0.05 being considered significant.

## Results

### Validation of tests comparing fasted and ad lib fed mice

**Figure 1:**
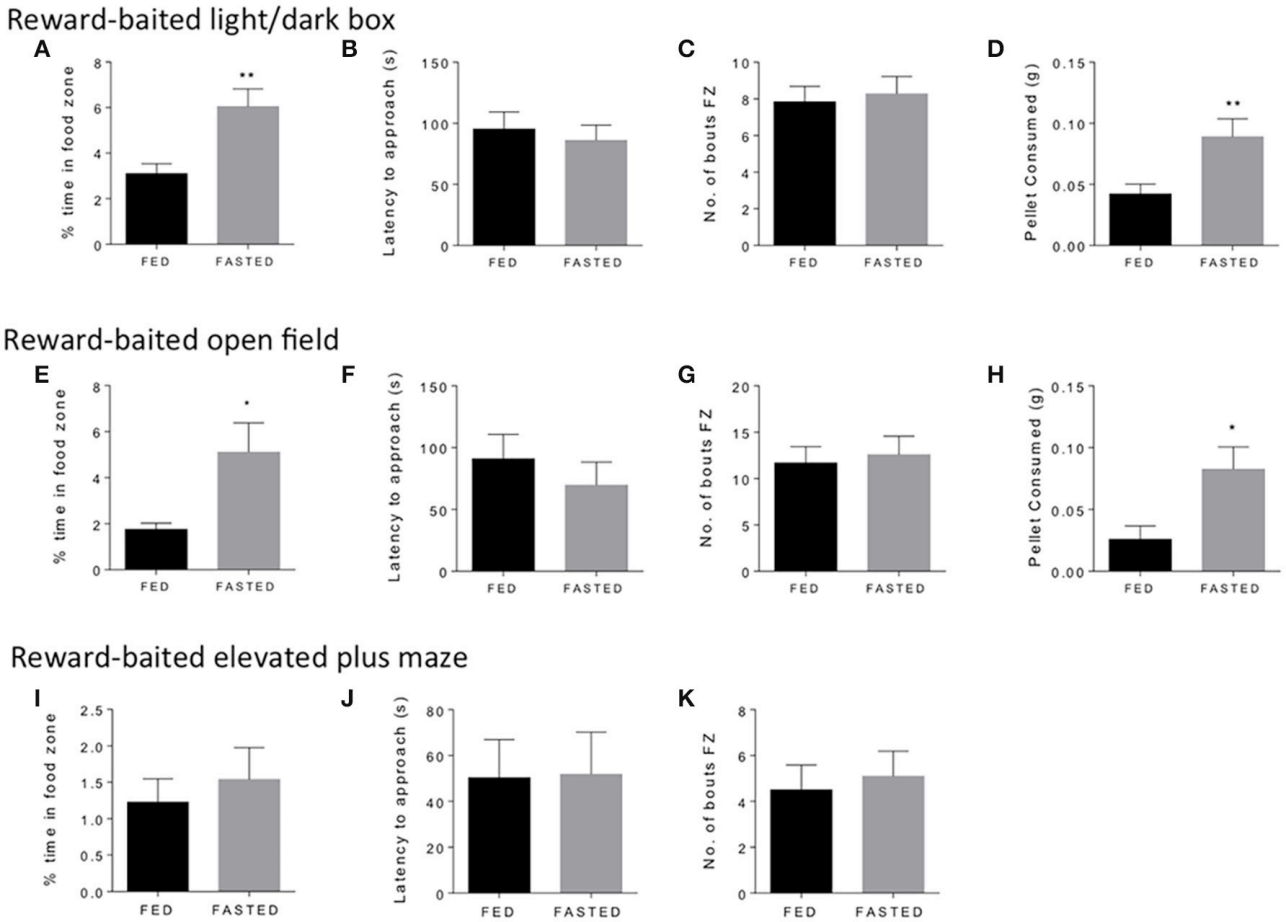
**Fasting increases time in food zone and consumption of peanut butter chip reward**. Comparison of *ad libitum* fed and 18 h fasted mice in the reward-baited light/dark box **(A–D)**, *n* = 18; reward-baited open field test **(E–H)**, *n* = 12; and reward-baited elevated plus maze, **(I–K)**, *n* = 20–25. FZ, food zone; ^*^p < 0.05, ^**^p < 0.01, independent measures *t*-test.

We started with three common tests of “anxiety” in mice, the open field, the light/dark box, and the elevated plus maze. We used fasted mice to validate the concept, as fasting is a well-established method to induce motivated responding and food intake (Toth and Gardiner, 2000). We considered a number of aspects of experimental design that might impact upon test performance. To this end, we performed our tests close to the end of the light cycle, when mice are naturally becoming more alert and hungry. We considered the distance between the safe zone and the food reward, which we felt needed to carefully balance the anxiogenic nature of the open zone, with the motivational pull of the PB chip reward. We found that in general, we needed group sizes of 12–15 mice to see significant results for time spent in the food zone and amount of PB chip consumed in the experiments we performed. Figure 1 shows the results of the three tests. Mice fasted for 18 h spent significantly more time in the food zone (Figures 1A,E) and ate significantly more of the PB chip (Figures 1D,H) in both the open field and the light dark box tests. We did not see any differences in latency to approach to food zone (Figures 1B,F) or number of bouts in the foods zone (Figures 1C,G) for either test. In the elevated plus maze, we did not see any differences in time spent in the food zone, which was overall very low, or in any other parameter (Figures 1I–K). Variability of results was particularly high for this test. We concluded that the elevated plus maze was a less suitable test for these modifications than the open field and light dark box, so did not pursue this test further. We then set out to validate our modified open field and light dark box test in additional experimental conditions of modified metabolic and motivational states.

### Ghrelin treatment increases PB chip consumption and time in food zone

**Figure 2:**
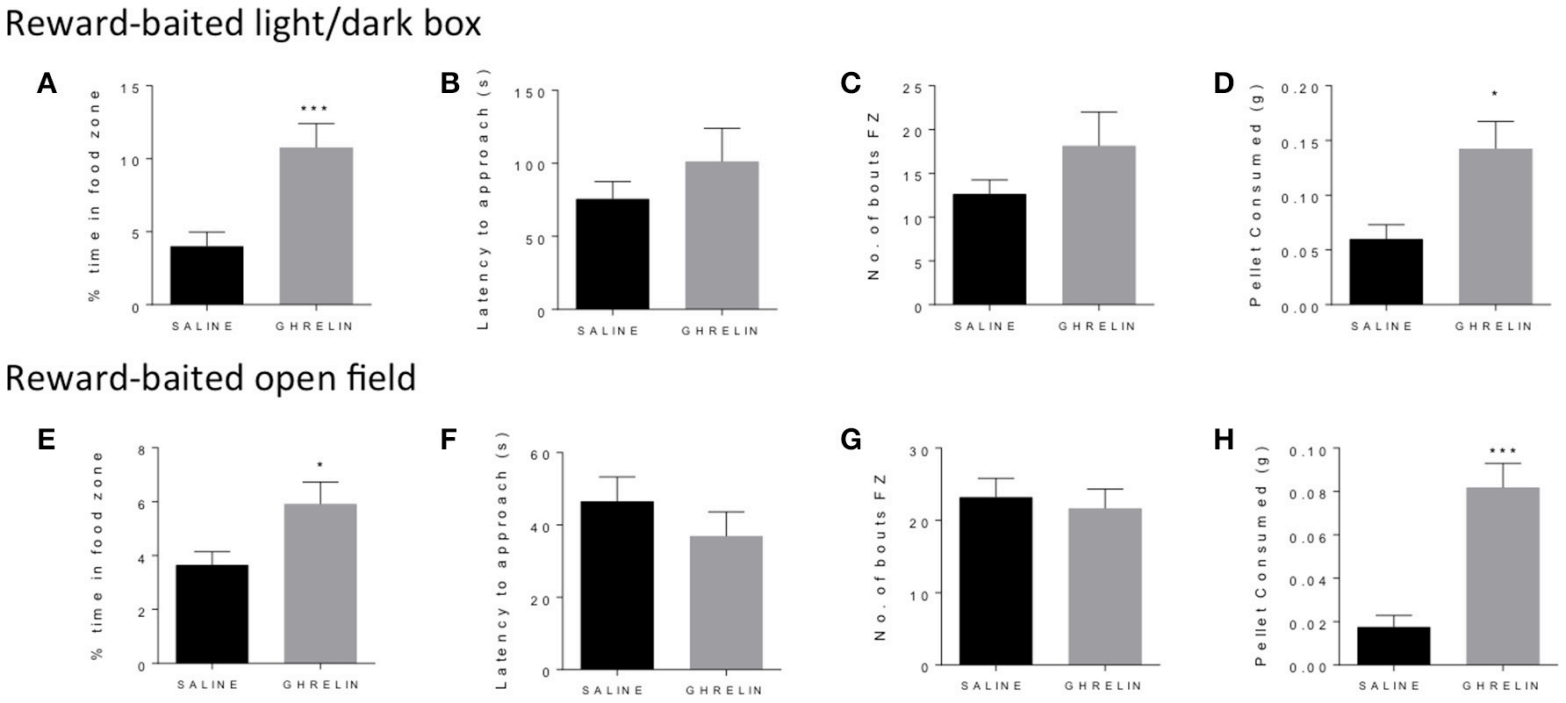
**Ghrelin treatment increases time in food zone and consumption of peanut butter chip reward**. Comparison of saline treated and ghrelin treated mice in the reward-baited light/dark **(A–D)**, *n* = 8; and reward-baited open field test **(E–H)**, *n* = 16–18. FZ, food zone; ^*^p < 0.05, ^***^p < 0.001, independent measures *t*-test.

Similarly to fasting, ghrelin has been well-described to increase both food consumption and motivated responding in operant tasks (Dickson et al., 2011). We administered ghrelin or saline to fed mice and repeated the baited open field and light/dark box as for the fasted mice. In both the light dark box (Figure 2A) and the open field (Figure 2E), we saw a significant increase in time spent in the food zone in the ghrelin-treated mice. This was supported by increased consumption of the PB chip in ghrelin treated mice (Figures 2D,H), in line with the well-known orexigenic actions of ghrelin. We did not see differences in latency to approach the food zone (Figures 2B,F) or number of bouts in the food zone (Figures 2C,G) in either test, suggesting that there were no differences in baseline anxiety-like behavior in ghrelin and vehicle-treated mice. Interestingly, we saw significant increase in locomotor activity in ghrelin-treated mice in the light/dark box only (Supplementary Figure 1C), which was not seen in the open field test.

### Glucose pretreatment reduces PB consumption and food zone time in fasted mice

**Figure 3:**
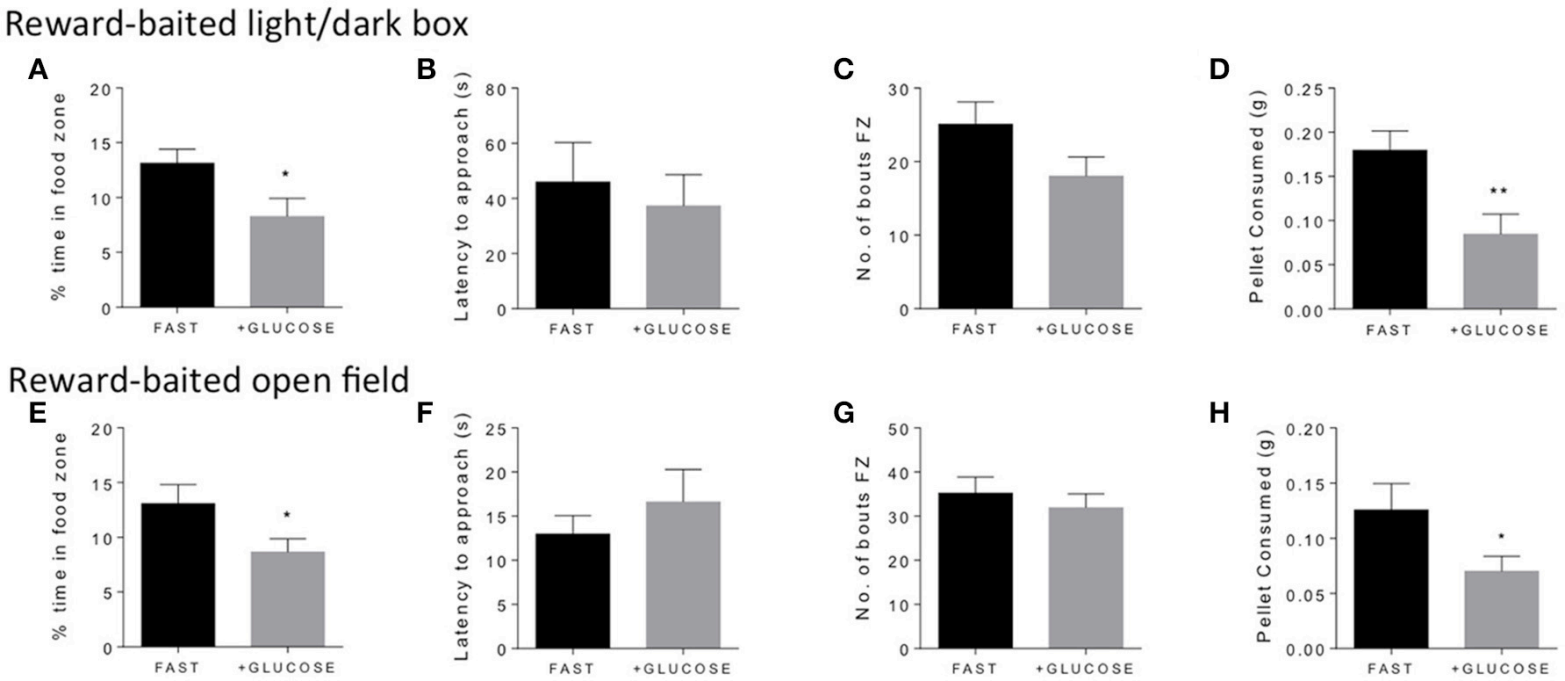
**Glucose treatment in fasted mice decreases time in food zone and consumption of peanut butter chip reward**. Comparison of 18 h fasted, saline treated, and 18 h fasted, glucose treated mice in the reward-baited light/dark **(A–D)**, *n* = 15–16; and reward-baited open field test **(E–H)**, *n* = 12–14. FZ, food zone; ^*^p < 0.05, ^**^p < 0.01, independent measures *t*-test.

In order to validate that energy status was driving the motivated behavior in the anxiogenic environment, we wondered if acutely mitigating the energy deficit in fasted mice with IP administration of glucose would reduce interest in the PB chip. We found that, indeed, giving glucose to fasted mice 10 min before testing significantly reduced the amount of time fasted mice spent in the food zone in both the light/dark box (Figure 3A) and the open field (Figure 3E). Additionally, it significantly reduced the amount of PB chip consumed in both tests (Figures 3D,H). Again, no differences were noted in either latency to approach (Figures 3B,F) or number of bouts in the food zone (Figures 3C,G).

Generally, we did not see differences in time spent in the anxiogenic (center of the open field or light side of the light dark box) or anxiolytic zones (perimeter zone of the open field or dark side of the light/dark box; Supplementary Figure 1). The exceptions to this were decreased time spent in the perimeter zone for fasted mice compared to fed mice in the open field (Supplementary Figure 1K) and increased time in the perimeter zone for fasted mice treated with glucose compared to fasted mice treated with saline (Supplementary Figure 1Q). Both these results reflect the increased time the fasted group spent in the food zone, and do not represent a true anxiety based result. With the exception of the already mentioned ghrelin-treated group we did not see alterations in locomotor activity between groups (Supplementary Figure 1).

## Discussion

Food consumption in laboratory rodents is almost exclusively measured in the context of *ad libitum* feeding in a home-cage environment. This fails to adequately model the reality of food seeking in a natural environment where attainment of food necessarily involves risk taking. We designed a series of experiments in which the mouse understands the value of the reward, but not the risk required to obtain the reward. To validate these tests, we manipulated the metabolic state to influence the decision to commit to food seeking in the novel, anxiogenic environment. We specifically set out to address a hole in the current battery of tests used to assess feeding and food reward, and attempted to design tests that would incorporate ecologically relevant risk processing, as currently available tests do this poorly (Anselme, 2015). To this end, we have described and validated two tests, which have innate perceived risk to the mouse, are sensitive to metabolic state, and are repeatable. These two tests are the reward-baited open field, the reward-baited light/dark box, which have the additional advantage of being cheap, time-effective and using standardized equipment readily available to most labs. We attempted to modify the elevated plus maze in a similar manner, by baiting the open arms with PB chips, but this test proved difficult to validate. It showed high variability within groups and low levels of investigation/consumption of the BP chips. We concluded the elevated plus maze apparatus was not appropriate for this modification to assess risk in food seeking.

We found no differences in time spent in the light zone of the light/dark box or center zone of the open field for any experimental condition, which demonstrates that mice explore the novel anxiogenic space similarly, regardless of metabolic state. This is an important parameter to measure to preclude the possibility that the experimental variable being tested influences anxiety state. Increases in anxiety will alter perceived risk and may confound assessment of the motivational/rewarding aspects of interaction with the PB chip. In two experiments, we report a difference in time spent in the perimeter zone of the open field, with the more metabolically replete (ad lib fed or glucose-treated) mice showing increased time in this space. This is reflective of the increased time the fasted mice spend in the food zone, and indicates greater motivation for the food reward rather than an altered anxiety state (Lockie and Andrews, 2013).

Locomotor activity was similar between all groups except for ghrelin-treated mice in the light/dark box. Ghrelin has previously been shown to increase locomotor activity in the absence of food (Jerlhag et al., 2006), although others have demonstrated that it has the opposite effect in the presence of food (Tang-Christensen et al., 2004). We might have expected, given the presence and increased consumption of the PB chip in this group, to have reduced locomotor activity in the ghrelin treated mice. We suggest that this result is due to an interaction between ghrelin signaling and the novelty and anxiogenic nature of the arena, as ghrelin has been shown to reduce anxiety in the face of stress (Spencer et al., 2012).

The results were most uniform in the ghrelin-treated experiment, and least uniform in the fasted mice treated with glucose, which likely reflects the greater innate physiological variability of the interventions. Ghrelin targets key neuronal populations that influence food seeking and motivation. Hypothalamic AgRP neurons are highly ghrelin sensitive and are active during fasting. Artificial activation of these neurons results in increased motivation to obtain food, increased consumption of food (Aponte et al., 2011; Krashes et al., 2011) and increased risk taking behavior around food acquisition (Jikomes et al., 2016).

One criticism of the current models of risk/reward decision making in mice is that the organism's survival is not endangered by bad choices, because at worst “bad” choices lead to loss of reward, not punishment (Anselme, 2015). Negative consequences, or at least concrete fear of them, is required to generate real awareness of risk within the organism, as is clearly demonstrated in food seeking and foraging choices undertaken in the wild (Holmes, 1984; Anderson, 1986). This can be done by punishment intrinsic to experimental procedure, or fear of natural consequences, like starvation, or predation. We used a larger than normal open field to exploit and exacerbate the natural fear of predation in open spaces (Holmes, 1984; Anderson, 1986), which underlies the traditional open field and light/dark box tests (Crawley, 2006), and force the mouse to make a calculated decision about the value of a known palatable food reward situated in the anxiogenic zone. This value ascribed to the PB chip following this assessment by the mouse can be termed the incentive salience of the reward, and should be sensitive to the metabolic state of the mouse. If this was the case, we would expect the lure of the PB chip to be greater in those mice experiencing metabolic need, and that these mice would make “riskier” choices about approaching and consuming the PB chip. Indeed, we demonstrated that hunger or exogenous ghrelin increased time spent eating and total amount consumed of the chip, while fasted mice injected with glucose immediately prior to the test behaved like fed mice. This indicates that the metabolic state of the mouse, while in the test situation, greatly influences its behavioral choices. Others have demonstrated that manipulation of AgRP neurons is capable of directing similar behavioral choices, driving food consumption in the presence of foot shocks (Jikomes et al., 2016) or directing more risky foraging behavior (Padilla et al., 2016). While we did not directly assess the role of AgRP neurons in our experiments, given that they are active in the fasted state (Yang et al., 2011), are targeted by ghrelin (Nakazato et al., 2001), and are glucose sensitive (Claret et al., 2007), we propose AgRP neurons are likely involved in mediating these behavioral changes.

Importantly, we do not see differences in other measures of exploratory or anxiety-like behavior such as time spent in anxiogenic zone or latency to approach PB chip. This demonstrates that we are not impacting these behaviors by altering metabolic state, but having a focused effect on consumptive behavior. Several times we saw a significant difference in time spent in the non-anxiogenic zone (open field perimeter zone or dark zone in light dark box), with the non-metabolically challenged mice showing increased time in this space. This is likely to be the reciprocal of the decreased time spent in the food zone during these tests. This indicates that mice are spending a similar amount of time exploring the anxiogenic zone, but without a pressing metabolic need to be in the food zone they prefer to spend time in the non-anxiogenic zone. This reflects the preference mice have for safer spaces when there is no incentive to leave them.

If we understand hunger, or high ghrelin, as a signal of food resource scarcity, the risk of starvation becomes more salient to the mouse than the risk of predation. In the fed state, there is no resource pressure as food has been constantly available. Fasting or administering ghrelin creates perceived resource pressure on the food supply, and amplifies the perceived consequences of forgoing the PB chip out of fear. Therefore these mice are more likely to spend time in the unsafe zone consuming a known high-energy food source, as the cost of forgoing this opportunity is greater than the fear of predation. Fed mice are much more conservative in their risk/reward calculation because they have no immediate metabolic pressure to contend with. We used highly palatable PB chips, however some experiments may require use of standard chow or other less well-liked food rewards to avoid ceiling effects on food exploration and consumption.

Others have used modified light/dark box to assess food consumption in an aversive environment (Teegarden and Bale, 2007; Cottone et al., 2012; Liu et al., 2016). Each of these previous uses of the modified light dark box comprise of a food reward placed in the light compartment and then investigated both time spent in the light compartment and time spent consuming food. An increased amount of time spent in the light compartment has universally been described as “risk-taking behavior,” however what consumption of the food under those circumstances means is more controversial. Cottone describes it as “compulsive-like eating” based on the premise that food intake is usually suppressed when a rat faces adverse circumstances like exposure in an open arena. However, arguing against “compulsive” eating is the fact that ad lib fed mice do not consume much of the PB chip, compared to fasted or ghrelin-treated mice. We feel that rather than representing “compulsivity” in fasted and ghrelin-treated mice, food consumption in an aversive environment is rather a logical response to a metabolic need, which is more pressing than the risky environment of the open arena. This paper offers a standardized and validated protocol for use of a reward-baited light/dark box, in addition to a reward-baited open field test. Using both tests in conjunction provides comprehensive assessment of risk vs. reward seeking in mice.

Food seeking in the modern human situation rarely has this perceived level of risk associated with it, none the less it is not risk free. Aside from the basic risks associated with driving or walking to the grocery store, there are social risks around food selection and consumption, which can make eating psychologically taxing. Perhaps the point here though, is not that we are modeling the human situation, but rather that we are trying to better model the natural food seeking-obtaining-consuming cycle of wild animals, which necessarily contains motivational, reward, and risk-taking elements. We propose that this test battery will be a useful addition to standard food intake measurements, and standard behavior tests to assess deficits in global processing of food attainment in genetic and other mouse models.

## Ethics statement

This study was carried out in accordance with the recommendations of Monash University Animal Ethics Committee guidelines. The protocol was approved by the Monash University Animal Ethics Committee (MARP 2014 026).

## Author contributions

ZBA, designed research and drafted manuscript. SHL, designed research, performed research, analyzed data, and drafted manuscript. CM, performed research and analyzed data. SR, performed research and analyzed data. NG, Performed research and analyzed data.

## Funding

This work was supported by fellowships from the Australian National Health and Medical Research Council to ZBA (1084344) and SHL (1072364).

### Conflict of interest statement

The authors declare that the research was conducted in the absence of any commercial or financial relationships that could be construed as a potential conflict of interest.
