# Supplementary figures and images for: Food Seeking in a Risky Environment: A Method for Evaluating Risk and Reward Value in Food Seeking and Consumption in Mice
Source: Front Neurosci. 2017 Jan 30;11:24. doi: 10.3389/fnins.2017.00024 (PMC5276994; doi:10.3389/fnins.2017.00024)

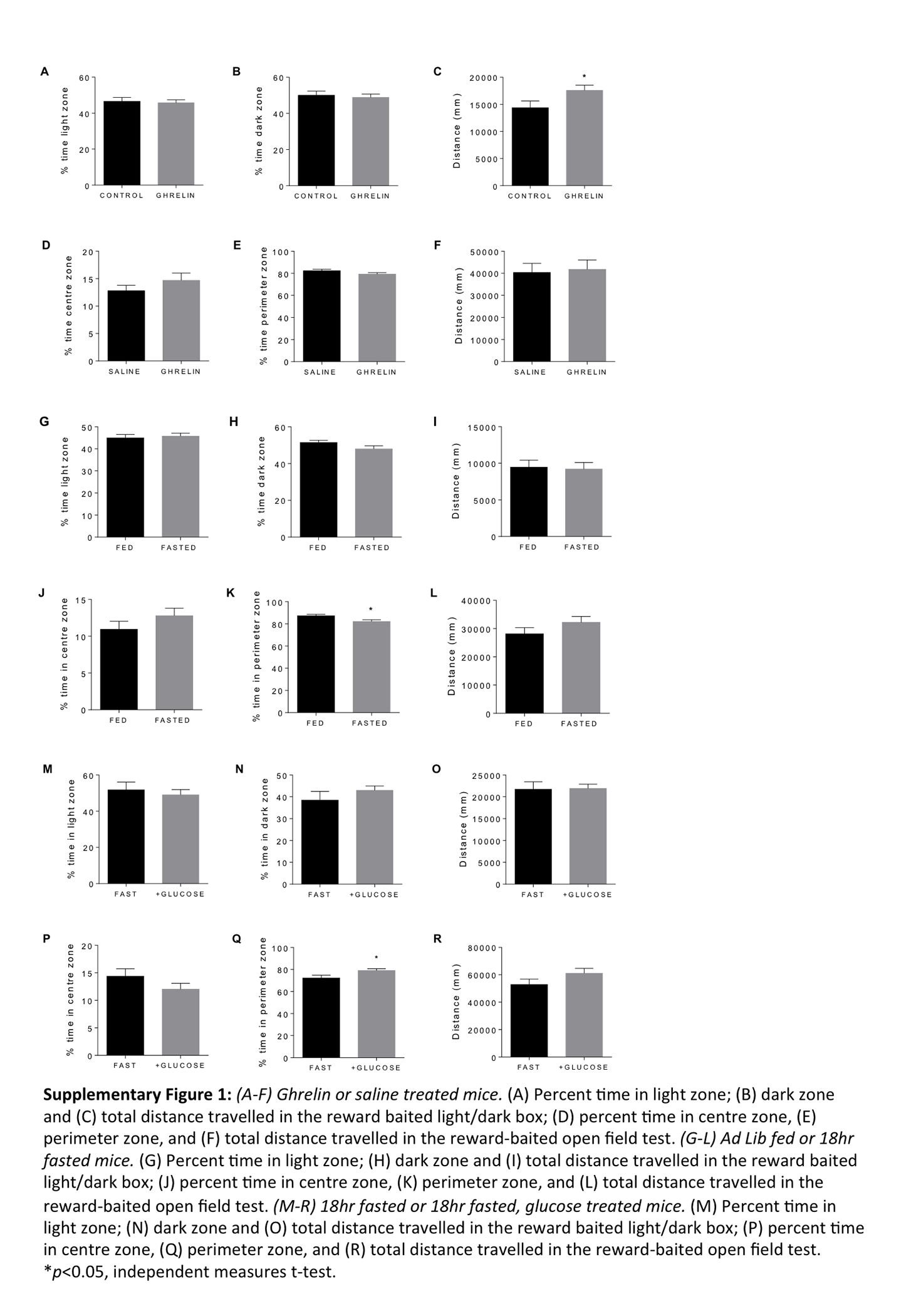

Supplement: Supplementary file 1 [file Image1.TIFF]

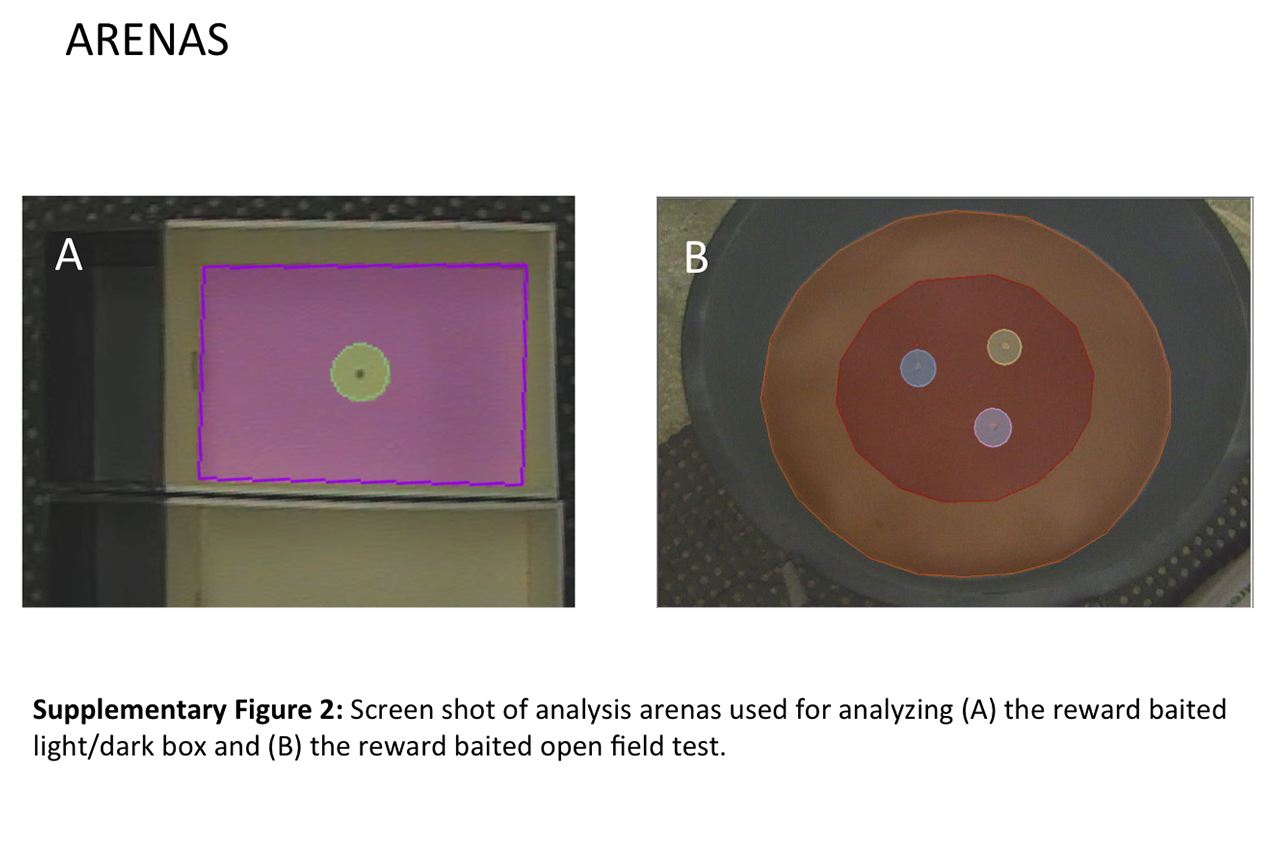

Supplement: Supplementary file 2 [file Image2.TIFF]
